# Long-Term Failure Rates of Interval Filshie Clips As a Method of Permanent Contraception

**DOI:** 10.1089/whr.2021.0017

**Published:** 2021-07-27

**Authors:** Kimberly A. Walhof, Lori M. Gawron, David K. Turok, Jessica N. Sanders

**Affiliations:** Department of Obstetrics and Gynecology, University of Utah, Salt Lake City, Utah, USA.

**Keywords:** Filshie, permanent contraception, sterilization failure, tubal sterilization

## Abstract

***Background:*** In 1996, the U.S. Collaborative Review of Sterilization (CREST) reported permanent contraception failure rates by method, but did not include the Filshie clip. Subsequent research provides data for Filshie clip failure rates up to 24 months, but rigorously designed and executed studies examining failure rates beyond 2 years are lacking.

***Objectives:*** To describe non-Filshie and Filshie procedures, identify failures, and calculate 10-year pregnancy rates among patients who have undergone interval permanent contraception procedures with Filshie clips.

***Study Design:*** We performed chart review for patients who underwent interval permanent contraception procedures between 2000 and 2014 at our institution. We identified births after permanent contraception by utilizing both chart review and the Utah Population Database. We report results from life table analysis, with censoring at failure, 49 years of age, or last observed date of service.

***Results:*** In this cohort of 693 patients, surgeons most commonly used Filshie clips for interval permanent contraception (*N* = 547, 78.8%). We classified pregnancies after Filshie clip procedures as verified (*n* = 4) or self-reported (*n* = 3). We obtained 5 years of data for 411 patients (59.3% of all permanent contraception procedures), and more than 10 years of data for 257 patients (37.1%). We calculated a cumulative 5- and 10-year pregnancy rate to be the same, including both verified and self-reported pregnancies, of 9.8 (95% confidence interval [CI] 4.1–23.3)/1000 women using Filshie clips. The 10-year rate of verified pregnancy is 2.8 (95% CI 1.0–15.7)/1000 women.

***Conclusion:*** Overall, long-term failure of Filshie clip interval permanent contraception procedures is infrequent, with a 10-year cumulative probability of failure of 4.1–23.3/1000 procedures performed. Filshie clips compare favorably with other methods of permanent contraception included in the CREST study, where the 10-year cumulative probability of failure ranged from 7.5 to 36.5/1000 procedures performed.

## Introduction

Female permanent contraception remains the most common method of contraception in the United States, with 18.6% of women aged 15–49 years reporting a permanent contraception procedure. Use of permanent contraception increases with age; among 20–29-year-old women, only 4.2% rely on female permanent contraception, while 39.4% of women age 40–49 years use this method.^1^ Timing of permanent contraception may be immediately postpartum or interval and can be achieved with a variety of approaches, including *via* laparotomy, laparoscopic, and hysteroscopic procedures. Methods of occlusion range from electrocoagulation to complete salpingectomy.

Counseling on the efficacy of female permanent contraception is frequently based on findings from the U.S. Collaborative Review of Sterilization (CREST), a multicenter prospective study of women undergoing tubal permanent contraception with 8–14 years of follow-up. Ten-year failure rates range from 7.5 to 36.5/1000 procedures depending on the method and timing.^2^ Unfortunately, Filshie clip failure rates are missing from the CREST study.

The Filshie clip method is a simple and relatively common method of interval permanent contraception first introduced in the United States in 1996.^3^ Research comparing Filshie clips with other methods of permanent contraception is limited and gaps in knowledge remain. In a series of two randomized-controlled studies comparing Filshie clips with Hulka clips, Dominik et al.^4^ found failure rates of 1.1 versus 6.9/1000 procedures at 12 months (*p* = 0.06). The respective values for a subset of women who underwent laparoscopic procedures were 9.7 versus 28.1/1000 procedures at 24 months (*p* = 0.16).

Another series of two randomized-controlled studies published the same year compared Filshie clips and tubal rings and found a failure rate of 1.7/1000 procedures at 12 months for both Filshie clips and tubal rings.^5^ However, rigorously designed and executed studies examining Filshie clip failure rates beyond 24 months are lacking. To address these gaps in the literature, we sought to compare patient and procedure characteristics between Filshie and non-Filshie methods of interval sterilization within a single institution, describe sterilization failures, and calculate a 10-year failure rate for Filshie clip procedures.

## Materials and Methods

We performed a retrospective chart review in the University of Utah Health System (UUHS). The University of Utah Institutional Review Board approved this study (IRB_00099431). We first identified all reproductive age women who had accessed the UUHS for health care between 2010 and 2014. We then narrowed to only those individuals who had undergone a tubal permanent contraception procedure *via* common procedure terminology (CPT) codes at any point between 2000 and 2014. We used this approach to ensure that those with procedures in the earlier years of 2000–2010 were still in the UUHS afterward in 2010–2014 for any pregnancies to be captured after sterilization. We initiated data collection in April 2017. We restricted our sample to women age 18–49 years at the time of the procedure and censored subjects at age 49. We also conducted a sensitivity analysis excluding individuals who had their procedures at 35 years of age or older to better compare with contraceptive efficacy studies where these patients would be excluded.

We included tubal permanent contraception by any method *via* the open (laparotomy), laparoscopic, and hysteroscopic routes. We excluded salpingectomies, hysterectomies, and immediate postpartum procedures. We extracted data directly from the permanent contraception operative report, including age, parity, timing, method, and complications. We reviewed Filshie clips procedure operative reports to determine whether the surgeon followed the manufacturer's instructions for application, which state that a second clip should be applied when the first clip transects the tube, or when there is “doubt about the placement or performance of the Filshie Clip.”^6^ In some cases, the surgeons applied two Filshie clips per side without documenting doubt of placement. In these cases, we assumed that this was their routine preference, rather than reinforcement of poorly applied initial clips. We classified this method as distinct from the standard Filshie clip permanent contraception procedure.

We reviewed the medical record for subsequent permanent contraception procedures or hysterectomy, and for subsequent reproductive technology procedures performed within the UUHS including *in vitro* fertilization (IVF) or tubal reanastomosis *via* CPT and ICD codes. We manually extracted any report of pregnancy after permanent contraception, and classified these pregnancies as self-reported or verified. We considered patients with a mention of pregnancy in the chart, without clinical confirmation, as self-reported. If a patient had a positive pregnancy test, we considered this to be clinical confirmation and the pregnancy was classified as a verified pregnancy. In addition, if a maternally linked birth or fetal death certificate was located in the state database, we consider it to be a verified pregnancy. For verified pregnancies, we reported the location as intrauterine, ectopic, or pregnancy of unknown location, and the outcome of intrauterine pregnancy as live birth, induced abortion, or spontaneous abortion. Among the pregnancies after permanent contraception procedures that we identified, we did not find any intrauterine fetal demises reported within the medical record or Utah Population Database.

To identify births that occurred outside the UUHS, we evaluated patients with interval permanent contraception procedures and cross-referenced them with the Utah Population Database to identify maternally linked birth and fetal death certificates occurring after the permanent contraception procedure date. The Utah Population Database includes vital records across the state and not limited to UUHS, but it does not include data related to surgery for tubal reanastomosis or IVF attempts, and it does not link induced abortion to other records. We conducted a manual chart review for all patients and extracted date of last observed services through April 30, 2017, to ensure adequate follow-up for all subjects included in the study.

We used descriptive statistics to explore demographic and procedural data. We used chi-square tests and Student's *t*-test to examine any differences between Filshie clip and non-Filshie permanent contraception procedures. We used life tables and Cox proportional hazard modeling to examine cumulative failure rates.

## Results

We identified 1742 permanent contraception procedures in the UUHS between 2000 and 2014. Interval permanent contraception represented 693 cases, of which the surgeon used Filshie clips according to the manufacturer's instructions^6^ in 547 (78.9%) cases. The non-Filshie group representing the remaining 146 permanent contraception procedures in the cohort includes the following: Filshie cases (*n* = 31, 4.5%) when providers applied two Filshie clips per side without a noted indication for the second clip, Essure^®^ (*n* = 51, 7.3%), electrocoagulation (*n* = 28, 4.0%), multiple methods (*n* = 25, 3.6%), Endoloop^®^ or laparoscopic Pomeroy (*n* = 7, 1.0%), surgiclips or not specified (*n* = 4, <1%).

We obtained 5 years of data for 411 patients (59.3% of all permanent contraception procedures), and more than 10 years of data for 257 patients (37.1%). The cohort had a mean age of 32.0 years (range 18–49). The median number of previous pregnancies was 3 (range 0–18), and the median number of living children was also 3 (range 0–10). Differences in Filshie clip patients and non-Fishie patients are described in Table 1. Filshie clip patients were on average younger (mean age 31.4 standard deviation [SD]: 6.3 compared with 34.2 SD: 6.8). We had a minimum of 5-year follow-up on 57.1% of procedures and 10-year follow-up on 33.5% of Filshie clip patients.

**Table 1.** Descriptives of Fishie and Non-Filshie Procedures

	Non-Filshie methods, *n* (%)	Filshie clips, *n* (%)	*p*
Age category			<0.001
18–24	20 (14)	126 (23)	
25–29	24 (16)	144 (26)	
30–34	35 (24)	119 (22)	
35–39	38 (26)	106 (19)	
40+	29 (20)	52 (10)	
Living children			0.002
0	16 (11)	26 (5)	
1	16 (11)	38 (7)	
2	52 (36)	156 (30)	
3	34 (24)	153 (29)	
4	19 (13)	91 (17)	
5+	6 (4)	62 (12)	
Provider training			<0.001
Attending alone	56 (38)	45 (8)	
Resident	90 (62)	498 (92)	
Advanced practice	0 (0)	1 (0)	
Complications			0.822
No	141 (97)	528 (97)	
Yes	4 (3)	17 (3)	
Year of surgery			<0.001
2000	4 (3)	16 (3)	
2001	5 (3)	48 (9)	
2002	11 (8)	38 (7)	
2003	7 (5)	44 (8)	
2004	6 (4)	42 (8)	
2005	3 (2)	49 (9)	
2006	2 (1)	43 (8)	
2007	4 (3)	40 (7)	
2008	10 (7)	39 (7)	
2009	14 (10)	22 (4)	
2010	16 (11)	25 (5)	
2011	14 (10)	33 (6)	
2012	20 (14)	38 (7)	
2013	19 (13)	39 (7)	
2014	11 (8)	31 (6)	
Years of follow-up			<0.001
<5	46 (32)	136 (25)	
5 to <10	63 (43)	154 (28)	
10+	37 (25)	257 (47)	
Total	146 (21)	547 (79)	

**FIG. 1.**
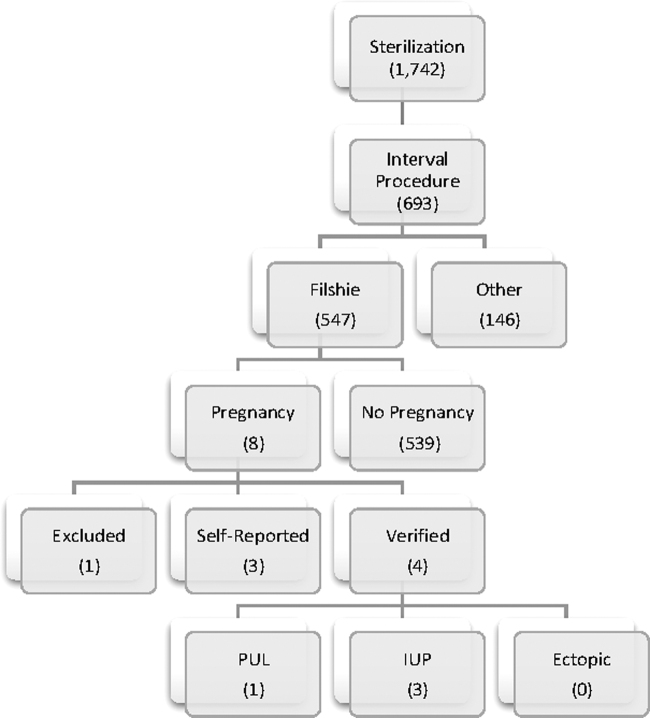
Outcomes of interval Filshie clip sterilization procedures.

Among 693 interval permanent contraception procedures, we identified 15 pregnancies (Fig. 1). A majority of the pregnancies were identified on initial chart review within the UUHS (*n* = 13), three of which were confirmed in the Utah Population Database. An additional two unique pregnancies were identified through the Utah Population Database. We attributed permanent contraceptive failures to 11 of 15 cases. The four nonfailures included a luteal phase pregnancy not identified at the time of the procedure and three pregnancies resulting from assisted reproductive technologies.

Of the remaining 11 pregnancies, 6 were self-reported to a health care provider, but without follow-up testing performed *or* with negative follow-up testing. In addition, we did not identify maternally linked birth or fetal death certificates corresponding to any of these cases. Three of the self-reports of pregnancy occurred after permanent contraception with Filshie clips, two occurred after Essure procedures, and one occurred after a cauterization procedure (Table 2). Of the six self-reported pregnancies, one seems likely to represent an actual pregnancy, three seem unlikely, and two are indeterminant based on the available records.

**Table 2.** Pregnancies in Utah Women After Interval Permanent Sterilization Procedure, 2000–2017

Method	Information source	Verified (yes or no)	Assisted reproductive procedure (yes or no)	Pregnancy outcome (only for verified pregnancies)
Filshie	UPDB, UUHS	Yes	Yes	Live birth
Filshie	UPDB, UUHS	Yes	No	PUL, spontaneously resolved
Electrocoagulation	UUHS	No	No	
Filshie	UUHS	No	No	
Filshie	UUHS	No	No	
Filshie	UPDB, UUHS	Yes	No	Live birth
Filshie	UUHS	No	No	
Filshie	UUHS	Yes	No	Termination
Electrocoagulation	UUHS	Yes	No	Salpingectomy for ectopic
Two Filshies per tube	UUHS	Yes	Yes (tubal reanastomosis)	Salpingectomy for ectopic after tubal reanastomosis
Essure	UUHS	No	No	
Two Filshies per tube	UUHS	Yes—Luteal phase pregnancy	No	Live birth
Essure	UUHS	No	No	
Filshie	UPDB	Yes	No	Live birth
Essure	UPDB	Yes	Yes	Live birth

PUL, pregnancy of unknown location; UPDB, Utah Population Database; UUHS, University of Utah Health System.

We identified five verified failures in the UUHS records and the Utah Population Database. Four pregnancies occurred after Filshie clip interval permanent contraception procedures and one after electrocoagulation. Of the four pregnancies that occurred after Filshie clips, three resulted in intrauterine pregnancies. Two live births resulted, and one pregnancy was terminated. The fourth pregnancy resolved without intervention and was labeled a pregnancy of unknown location.

We calculated the 10-year failure rate for Filshie clip procedures, including self-reported pregnancies, as a cumulative probability per 1000 procedures performed. We also conducted a sensitivity analysis with only verified failures. When including all self-reported pregnancies, the cumulative probability of failure after 1 year is 3.8 (95% confidence interval [CI] 0.9–15), after 2 years is 7.7 (95% CI 2.9–20.4), and from 3 to 10 years is 9.8 (95% CI 4.1–23.3)/1000 procedures performed. When including only verified pregnancies, the cumulative probability of failure after 1 year is 1.9 (95% CI 0.3–13.3). The probability does not increase after 2 years, and then from 3 to 10 years the cumulative probability of failure is 2.8 (95% CI 1.0–15.7)/1000 procedures performed (Table 3). In the sensitivity analysis, excluding 225 individuals who were 35 years or older at the time of procedure, cumulative failure was slightly higher than the full sample, with a 10-year cumulative probability of failure, 13.6 (95% CI 5.7–32.4)/1000 procedures.

**Table 3.** Cumulative Probability of Pregnancies Among Women Undergoing Interval Tubal Permanent Contraception *Via* Filshie Clips, Per 1000 Procedures (95% CI), *N* = 547

	Years since Filshie clip procedure and follow-up (*n*)									
	1 (514)	2 (497)	3 (466)	4 (410)	5 (359)	6 (323)	7 (291)	8 (258)	9 (207)	10 (165)
All pregnancies	3.8 (0.9–15.0)	7.7 (2.9–20.4)	9.8 (4.1–23.3)	9.8(4.1–23.3)	9.8 (4.1–23.3)	9.8 (4.1–23.3)	9.8 (4.1–23.3)	9.8 (4.1–23.3)	9.8 (4.1–23.3)	9.8 (4.1–23.3)
Verified pregnancies	1.9 (0.3–13.3)	1.9 (0.3–13.3)	2.8 (1.0–15.7)	2.8 (1.0–15.7)	2.8 (1.0–15.7)	2.8 (1.0–15.7)	2.8 (1.0–15.7)	2.8 (1.0–15.7)	2.8 (1.0–15.7)	2.8 (1.0–15.7)

CI, confidence interval.

## Discussion

We found that failure of Filshie clip interval permanent contraception procedures is infrequent and comparable with other methods, including in those younger than 35 years at the time of procedure. Previous research demonstrated that short-term failure rates at 12 months range from 1.7 to 3.9/1000 procedures in the available literature, which includes only two randomized-controlled trials.^4,5^ Only one publication includes a 24-month follow-up, with a failure rate of 9.7/1000 procedures.^4^ Filshie clips compare favorably with other methods of permanent contraception included in the CREST study, where the 10-year cumulative probability of failure ranged from 7.5 to 36.5/1000 procedures performed. These results can reassure both patients and providers in their choice of sterilization method. While a rigorous randomized-controlled trial would be cost prohibitive for an established sterilization method, a prospective observational trial with follow-up comparative with the CREST study would allow for a more precise failure estimate.

There are limitations to our study. First, the CREST study was prospective and included periodic follow-up with patients by phone to establish permanent contraception failure rate. We attempted to overcome the limitation of retrospective chart review by including only patients who had recent care within the UUHS, and by using the Utah Population Database to capture maternally linked births or fetal demise across the state of Utah. However, we may have missed pregnancies reported to providers outside of the UUHS, IVF or reanastomosis performed outside of UUHS, pregnancies that occurred in patients who temporarily or permanently left Utah, or pregnancies terminated or spontaneously resolved without interaction in the UUHS. Another limitation is our sample size. With only 547 Filshie clip interval tubal procedures in our chart review, our sample size is smaller than most methods included in the CREST study, however, CIs are narrow suggesting these numbers are sufficiently powered for comparative purposes.

In conclusion, tubal permanent contraception can be achieved by many methods, including the Filshie clip, a simple and easy device to use. This retrospective chart review suggests that interval permanent contraception with Filshie clips compares favorably with other methods of tubal permanent contraception described in the CREST study. The retrospective Filshie clip data presented here do not serve as a direct comparator with the prospective CREST data.

## Abbreviations Used

CI: confidence interval
CPT: common procedure terminology
CREST: Collaborative Review of Sterilization
IVF: *in vitro* fertilization
SD: standard deviation
UUHS: University of Utah Health System
